# Epidemiology and Prevalence of Musculoskeletal Disabilities Following Motor Vehicle Accidents in Aljouf Region, Saudi Arabia

**DOI:** 10.3390/medicina60101562

**Published:** 2024-09-24

**Authors:** Dalia Mahmoud Abdelmonem Elsherbini, Lashin Saad Ali, Nesma M. Allam, Radwa T. Elshorbagy, Hadaya Mosaad Eladl, Ateya Megahed Ibrahim, Yasser M. Elbastawisy, Mamdouh Eldesoqui, Mohamed El-Sherbiny

**Affiliations:** 1 Department of Clinical Laboratory Sciences, College of Applied Medical Sciences, Jouf University, P.O. Box 2014, Sakaka 72388, Saudi Arabia; 2 Department of Basic Medical Sciences, Faculty of Dentistry, Al-Ahliyya Amman University, Amman 19111, Jordan; l.lashin@ammanu.edu.jo; 3 Department of Medical Physiology, Faculty of Medicine, Mansoura University, Mansoura 35516, Egypt; 4 Department of Physical Therapy and Health Rehabilitation, College of Applied Medical Sciences, Jouf University, P.O. Box 2014, Sakaka 72388, Saudi Arabia; nmallam@ju.edu.sa (N.M.A.); radwa_pt@yahoo.com (R.T.E.); hd.mos@ju.edu.sa (H.M.E.); 5 Department of Physical Therapy for Surgery, Faculty of Physical Therapy, Cairo University, Giza 11432, Egypt; 6 Department of Physical Therapy for Musculoskeletal Disorders and Their Surgeries, Faculty of Physical Therapy, Cairo University, Giza 11432, Egypt; 7 College of Nursing, Prince Sattam bin Abdulaziz University, Al-Kharj 11942, Saudi Arabia; a.eleglany@psau.edu.sa; 8 Department of Family and Community Health Nursing, Faculty of Nursing, Port Said University, Port Said 42511, Egypt; 9 Department of Basic Medical Sciences, College of Medicine, Al-Rayan Colleges, Al-Madinah 41311, Saudi Arabia; 10 Department of Basic Medical Sciences, College of Medicine, AlMaarefa University, P.O. Box 71666, Diriyah 11597, Saudi Arabia; mamrah@um.edu.sa (M.E.); msharbini@um.edu.sa (M.E.-S.); 11 Department of Anatomy, Faculty of Medicine, Mansoura University, Mansoura 35516, Egypt

**Keywords:** disability, fracture, traumatic amputation, traffic accidents, injury severity score, Saudi Arabia

## Abstract

*Background and Objectives*: Motor vehicle accidents (MVAs) are the leading cause of disability, particularly among young adults in Saudi Arabia. Persistent disabilities account for around 7% of all injuries attributed to MVAs in Saudi Arabia in the last twenty years. Limited studies on musculoskeletal disabilities following MVAs have been carried out in Saudi Arabia. This study aims to explore the epidemiology and prevalence of musculoskeletal disabilities in motor vehicle accident (MVA) patients in the Aljouf region, Saudi Arabia. *Materials and Methods*: This retrospective cross-sectional study evaluated all MVA victims treated in the Aljouf region, Saudi Arabia, from January 2020 to December 2022. A total of 3252 medical records were collected, with 731, 1197, and 1324 musculoskeletal injury cases per year, of which 88, 168, and 153 records from 2020, 2021, and 2022 were analysed, respectively. *Results*: The study found that patients aged 25–34 and 35–44 years were the most likely to experience disability following MVAs. The difference between age groups during a single year was statistically significant (*p* < 0.001). Most patients were male (89.8%, 82.7%, and 79.7%) during 2020, 2021, and 2022, respectively. The majority of injuries involved the upper extremities (38.6%, 36.9%, and 40.5%), followed by lower extremities (36.4%, 35.7%, and 34.6%), head and neck (21.6%, 26.2%, and 34.6%), thoracic region (20.5%, 24.4%, and 17%), and finally lumbosacral spine (6.8%, 5.4%, and 6.5%) during 2020, 2021, and 2022, respectively, with a significant difference for each year (*p* < 0.001). The study found a link between the likelihood of developing high disability grades and injury severity scores. The patients with very severe ISS ≥ 25 (OR: ∞ CI 95%: 39.81–∞; *p* < 0.001), severe ISS = 16–24 (OR: ∞ CI 95%: 20.90–∞; *p* < 0.001), and moderate ISS = 9–15 (OR: ∞ CI 95%: 1.2–∞; *p* = 0.02) were at greater risk of developing high grades of disability. *Conclusions:* This study highlighted the musculoskeletal disabilities in the Aljouf region following MVAs. Severe musculoskeletal disabilities were rare, but fractures were the most common following MVAs. More efforts should be directed towards education on early transportation and transfer to the nearest medical centres, seeking assistance immediately after MVAs for patient safety, and disability prevention.

## 1. Introduction

Motor vehicle accidents are a significant public health concern, causing 1.35 million deaths and 50 million injuries annually, according to the World Health Organization [1]. These injuries often lead to impairments, potentially resulting in incapacity and potentially disability. As per the WHO, disability is more than just a health issue; it affects 15% of the global population and is influenced by bodily characteristics and societal environment [2]. Motor vehicle accidents are the primary cause of disability, especially among young adults, in Saudi Arabia [3]. Approximately 7% of motor vehicle accident (MVA) injuries in Saudi Arabia during the last 20 years have led to long-lasting disabilities [4]. Long-term rehabilitation is crucial for musculoskeletal deformities like spinal cord injury (SCI), amputation, lower extremity fracture, multiple fractures, or polytrauma [3].

Saudi Arabia, a country with one of the highest rates of traumatic SCI in the world, has conducted limited research on SCI compared to Western countries [5,6]. Cervical and thoracic/lumbar spinal cord injuries result in tetraplegia and paraplegia, affecting the musculoskeletal system, leading to muscle mass loss, atrophy, and reduced bone mineral density [7,8,9]. Motor vehicle accidents have been considered the leading cause of SCI, accounting for approximately 39.5% of all cases [10]. According to the World Bank and the WHO, the mortality rate of MVAs in Saudi Arabia reached 36 per 100,000 people in 2019, with a worldwide rate of approximately 17 per 100,000 people [11]. Notably, when compared with other injury causes, traumatic spinal cord injuries (TSIs) caused by MVCs showed the highest morbidity and mortality rates [12]. A study conducted by Alawad et al. [13] was the first epidemiological study concerning TSIs in the Kingdom of Saudi Arabia, stating that the lack of previous studies in the region made it difficult to compare the findings to others. The accurate incidence of spinal cord injuries (SCIs) in Saudi Arabia is limited due to limited information [6]. A hospital-based study reported an incidence rate of 2.1 per million between 2003 and 2008 [5], while an older study reported 377 patients sustained traumatic injury from 1979–1984 [6]. No published data exist on the prevalence and incidence of SCI [5,14]; however, over the last decade, in a project submitted by Abo Abad in 1999, the annual incidence of SCI was revealed to be 62.37 per million, with an increase from 1990 to 1994, and peaking in 1994 [15]. Another study submitted by Al-Shammari to the University of Birmingham, Birmingham, UK, showed an incidence of 38 per million, including traumatic and non-traumatic SCI, during 2000–2010 [16].

Hou et al. [17] reported that MVAs are frequently responsible for fractures, with the lower limbs being the most common site, followed by the upper limb, skull, and maxillofacial region. Fractures are a common consequence of MVAs, often resulting in significant disability and accompanied organ injuries [18]. The majority of MVA casualties in Saudi Arabia consist of young men who suffer from head injuries, fractures in the lower limbs, and various fractures in the upper limbs [4]. MVAs often lead to polytrauma, resulting in a higher abbreviated injury scale (AIS) score, resulting in more extended hospital and intensive care stays [19]. It is crucial to comprehend this problem in MVA patients, as it could indicate the necessity for extended rehabilitation [3].

In contrast to Western countries, a few articles on orthopaedic injuries in the kingdom were found in the literature. A study conducted by Alotaibi et al. [20] concerning the pattern of orthopaedic injuries among victims of road traffic accidents in the Aseer region, Saudi Arabia, revealed that localisation of reported injuries is highest for the lower limb, followed by the upper limb, pelvis, spine, and finally the head and neck region. The type of injury reported is highest for simple, complex, and open fractures, where the majority of patients recovered from their injuries, while a few had permanent disabilities. Al Nefaie et al. [21] systematically reviewed fracture patterns in Saudi Arabian road traffic accidents between 2010 and 2022, examining 11 studies [20,22,23,24,25,26,27,28,29,30,31] that collected medical record data on fractures due to road traffic accidents and enrolled 4079 patients. The regions selected in Saudi Arabia were Aseer, Riyadh, Al Kharj, Tabuk, Buraydah, and Almadinah. Ten studies were cross-sectional and collected data retrospectively, while only one was of a cohort design. 

This study is the first of its kind in the Aljouf region, aiming to investigate the prevalence of musculoskeletal disabilities following motor vehicle accidents and the epidemiology of associated injuries in these patients. We hypothesised that the data obtained from this study may guide planning and implementing primary preventive measures to reduce motor vehicle accident-induced musculoskeletal disabilities and improve health outcomes.

## 2. Materials and Methods

### 2.1. Study Design and Settings

This retrospective cross-sectional study evaluated all MVA victims treated in the Aljouf region at Prince Mutaib Bin Abdulaziz Hospital in Sakaka, Saudi Arabia, from January 2020 to December 2022. Prince Mutaib Bin Abdulaziz Hospital was designated a trauma centre in the Aljouf region.

### 2.2. Eligibility Criteria

The study population included adults aged ≥18 years, where age was categorised according to Shults et al. [32] of either sex, presenting with musculoskeletal disabilities due to MVAs, and attending the hospital. Study participants also included patients who attended the emergency department. Exclusion criteria were patients <18 years old and musculoskeletal injuries not resulting from MVAs.

### 2.3. Study Sample

The necessary sample size was determined based on the lack of information regarding the frequency of musculoskeletal disability in patients involved in road traffic accidents (RTAs) [33]. The sample size was calculated using the formula for proportions (binomial) [34]

*n* = *p* × (1 − *p*) × (*z*/*e*)^2^

where *n* is the sample size required for a large population, *p* is the proportion of post-RTA musculoskeletal disability, *z* is the confidence level, and *e* is the margin of error. *p* is based on previous studies showing that about 5.2–11.2% of patients have some degree of musculoskeletal impairment post-RTA [35,36,37], *z* is defined as 1.96 (for a 95% confidence level [α]), and *e* at 5%. With these considerations, the sample size is calculated to be 76–153, which indicates that this is an adequate sample size for estimating the proportion of musculoskeletal disability. The numbers of patients selected in the study were 88 in 2020, 168 in 2021, and 153 in 2022.

### 2.4. Data Collection 

**Figure 1:**
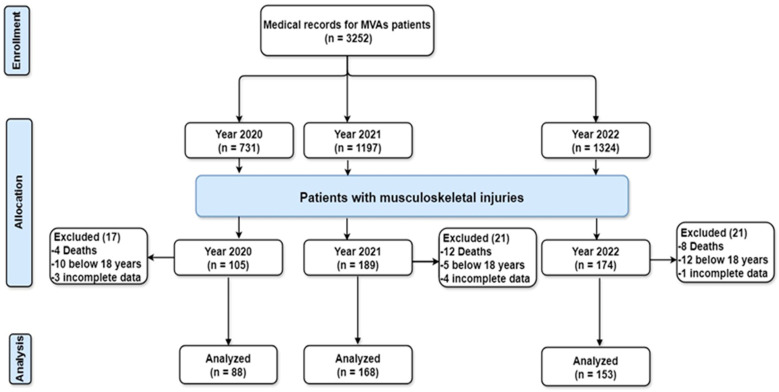
Study flowchart, MVAs: motor vehicle accidents.

A total of 3252 medical records were retrieved from the Prince Mutaib bin Abdulaziz Hospital registry system for patients with musculoskeletal injuries in 2020, 2021, and 2022. The data included demographic details (age, gender, and citizenship), initial assessment and treatment provided (initial Glasgow coma scale, location of injury, diagnosis, and status of hospital admission or discharge). The final number of medical records (88, 168, and 153, respectively) from the three years that met the inclusion criteria were analysed (Figure 1).

### 2.5. Injury Severity Score (ISS)

Patients were categorised based on injury location and extent. The severity of the injuries was determined using the abbreviated injury scale (AIS). The AIS describes the anatomic injury with a range of minor (1 point), moderate (2 points), serious (3 points), severe (4 points), critical (5 points), to incompatible with life (6 points) in all five main body regions [38]. The injury severity score (ISS), a scoring system which is commonly used in traumatology [39], has values ranging from 0 to 75 and increases with severity; the higher the score, the higher the injury severity, and, therefore, the higher the mortality. An AIS score is assigned to each injury to establish the ISS, and only the highest AIS score in each body region is used to calculate the ISS. Scores corresponding to the three body regions with the most severe injuries are squared and added to obtain the ISS; therefore, the ISS consists of the sum of the squares of the highest degrees of AIS of each of the three body regions which have suffered the most severe injuries. In the case of a level 6 injury, an ISS of 75 is automatically assigned to the patient. A higher score indicates a more severe injury, with scores of 1–8 classified as minor, 9–15 as moderate, 16–24 as severe, and 25–75 as very severe [40].

### 2.6. Disability Score (DS)

The clinical disability score was used due to the large sample size and the challenge of following up with all the patients. This score employed a five-point scale (0–4) as previously described by Bull [41]. A score of “0” indicates that the person can return to everyday life without any noticeable or lasting disability. A score of “1” showed a slight disability that allows for a full range of activities but not a complete return to normality. A score of “2” indicated a moderate disability that may affect certain activities, such as sports. A score of “3” indicated a severe disability that would limit normal activities and potentially preclude special activities. A score of “4” indicated a very severe disability that would render a person mainly or incapable of their everyday activities. For example, slight “1” disabilities include moderate painless loss of movement in a joint or scarring elsewhere than the face; moderate “2” disabilities include some instability of a knee, shortening of a lower limb by about 1 inch (2.5 cm), amputation of digits, facial scarring, speech defects, deafness in one ear, or the loss of sense of smell. Severe “3” disabling injuries included major amputations, gross limitation of movement of major joints with pain, severe brachial plexus lesions, or loss of sight of an eye. Very severe “4” disabilities included substantial and irreversible brain damage or spinal cord damage with paraplegia.

### 2.7. Statistical Analysis

The study used IBM SPSS version 25 for data analysis, presenting quantitative data as mean ± standard deviation and qualitative data as percentages. The chi-square test was used for proportion comparison, while the Kruskal–Wallis test was used for continuous variables comparison. Odds ratios were used to assess associations between the dependent variable (disability scores 3 and 4) and other variables, with a significance level *p*-value < 0.05. Odds ratios were interpreted as low 1.44, medium 2.48, and high 4.27 [34].

## 3. Results

A total of 409 medical records were obtained from the Prince Mutaib bin Abdulaziz Hospital registry system upon meeting the inclusion criteria during the years 2020–2022. The mean ± SD age of patients was 34.86 ± 10.32, 34.27 ± 12.22, and 33.97 ± 12.19 years for 2020, 2021, and 2022, respectively, with no significant difference. The prevalence of disability varied among age groups, with the lowest rates found among a certain age (≥65 years) and highest among the age group 25–34 years (2020 and 2022 years), but during the 2021 year, the age group 35–44 years was the most prevalent. The difference between age groups during a single year was statistically significant. The majority of the patients were male (89.8%, 82.7%, and 79.7%) during 2020, 2021, and 2022, respectively, revealing a decline in number with the years but still a significant difference compared to females. Most of the patients were Saudi (73.90%, 78.6%, and 78.4%) during the three years, with a significant difference compared to non-Saudi patients (Table 1).

**Figure 2:**
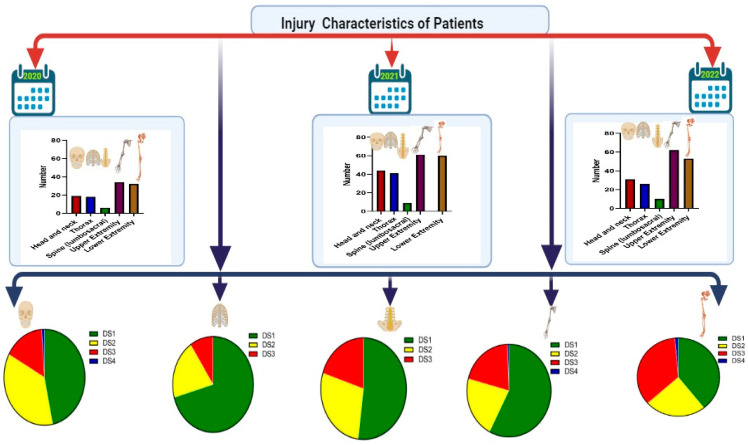
Injury characteristics of patients and prevalence of disability according to site of injury, DS: disability score.

Table 2 and Figure 2 show that single injuries were the most common during 2020, 2021, and 2022 (79.6%, 77.4%, and 85.0%), respectively, with a significant difference compared to two, three, or more injuries each year. It was observed that the percentage of two, three, or more injuries declined over the years. The majority of injuries involved the upper extremities (38.6%, 36.9%, and 40.5%), followed by lower extremities (36.4%, 35.7%, and 34.6%), head and neck (21.6%, 26.2%, and 34.6%), thoracic region (20.5%, 24.4%, and 17%), and finally lumbosacral spines (6.8%, 5.4%, and 6.5%) during 2020, 2021, and 2022, respectively, with a significant difference for each year but no significant difference between years. Analysis of injury severity score (ISS) showed that the mean ±SD score of patients was 11.82 ± 7.71, 11.23 ± 6.42, and 12.16 ± 7.28 during 2020, 2021, and 2022, respectively, which was non-significant between years. Patients with moderate ISS were most prevalent, followed by mild, severe, and very severe ISS, with significant differences in ISS scores each year. Disability score grade 1 was the most prevalent, followed by DS2, DS3, and DS4. Hospital admission percentage was high compared to discharged cases, with the most significant difference in 2021 (94%). Glasgow coma score (GCS) was not affected in injured patients.

The analysis of head and neck region injuries revealed that general head trauma is the most common complaint (50%, 72%, and 88.9%), with the skull base with temporal bone being the most affected region (50%, 12%, and 33.3%) during the three years. The nasal bone was also the most affected (63.6%, 25.0%, and 45.1%). Subaxial cervical fracture is more common than upper cervical fracture (C1–C2), which was non-significant between the years (Table 3).

The most common rib fractures in the thoracic region were found in the middle ribs (4th–9th) and lower ribs (10th–12th), which were non-significant between the years. Multiple rib fractures were the most common type, followed by single or double rib fractures. Right-sided rib fractures were the most common (83.3%, 85.2%, and 84.4%) during the 2020, 2021, and 2022 years, respectively. The last thoracic vertebra was the most liable for fracture, which was non-significant (Table 4).

Regarding the lumbosacral spine, the first (83.3%, 33.3%, and 57.1%) and second (42.9%, 33.3%, and 16.7%) lumbar vertebrae were the most affected, respectively, during the 2020, 2021, and 2022 years (Table 5).

The study revealed that the right side is the most common for upper limb injuries, with a significant difference between 2020, 2021, and 2022. Long bone fractures were the most common, with the humerus being the most common, followed by the radius, ulna, and clavicle. Dislocations were also common for the shoulder, elbow, carpometacarpal, and interphalangeal. Severe injuries, such as distal forearm amputation (2.9% in 2021) and quadriplegia (2.3% and 1.4%) during the years 2020 and 2022, respectively, were the least common injuries (Table 6).

Individual lower limb injury analysis revealed that the most common side to be injured is the right side (57.9%, 91.7%, and 62.7%) as compared with the left one (42.1%, 8.3, and 37.3%), with a significant difference during the 2020, 2021, and 2022 years, respectively. Long bone fractures were the most common type of injury, with the femur being the most prevalent, followed by the tibia and fibula. Long bone fractures were followed by pelvic bone fractures, like those of the acetabulum and hip bone. Metatarsal bones are more likely to be fractured and dislocated compared to tarsal bones. Dislocation is less common for the pelvis and interphalangeal compared to the upper limb. Severe injuries like lower limb amputation (2.4% in 2020) and quadriplegia (2.3% and 1.4%) were the least common types of injuries associated with the upper limb (Table 7).

Table 8 shows the prevalence of disability according to demographics and site of injury of patients. In terms of age group, no significant link was present between the age of patients and grade of disability; patients in the age group 25–34 and 35–44 years showed the highest number with DS1, while patients in age groups 25–34 showed highest number with DS2, DS3, and DS4. Regarding gender, there is no significant link between gender and developing disability, but the male gender exhibited the highest number in terms of disability grades. Patients with upper extremity injuries showed the highest number with DS1. In contrast, patients with lower extremities showed the highest number of DS2, DS3, and DS4 compared to other sites (Figure 2).

**Figure 3:**
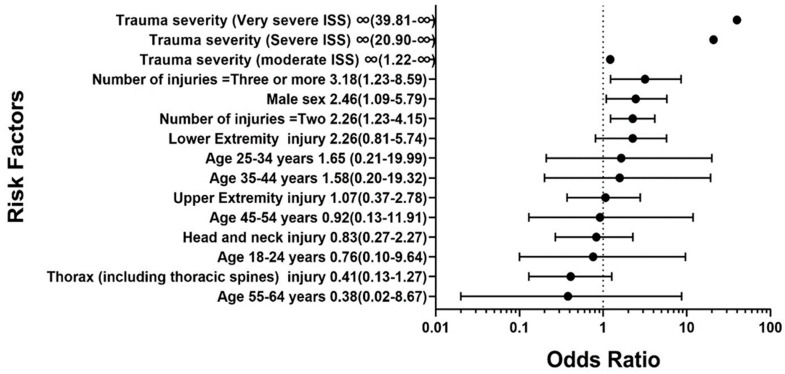
Forest plot for disability DS3 and DS4 risk factors ((odds ratio (95% CI)).

Table 9 and Figure 3 show the link between the likelihood of developing high grades of disability (DS3 and DS4) and the demographic and injury characteristics of the patients. In the sense of trauma severity (ISS), the patients with very severe ISS ≥ 25 (OR: ∞ CI 95%: 39.81–∞; *p* < 0.001), severe ISS = 16–24 (OR: ∞ CI 95%: 20.90–∞; *p* < 0.001), and moderate ISS = 9–15 (OR: ∞ CI 95%: 1.2–∞; *p* = 0.02) were at greater risk of developing high grades of disability. Regarding sex, there is a significant link between male gender and developing disability *(p* = 0.03). The number of injuries had a significant impact on developing high grades of disability. Patients with three or more injuries (OR: 3.18 CI 95%: 1.23–8.59; *p* = 0.03) and two injuries (OR: 2.26 CI 95%: 1.23–4.15; *p* = 0.03) were at high risk of developing high grades of disability. No significant relation was seen between the age of patients and developing a high grade of disability. Patients aged 25–34 were at higher risk (OR: 1.65, 95% CI: 0.21–19.99; *p* = 0.99) compared with other age groups. Patients with lower extremity injuries (OR: 2.26, 95% CI: 0.81–5.74; *p* = 0.16) were at the greatest risk of developing a disability compared with the other sites, but this was not significant.

## 4. Discussion

Road traffic accidents (RTAs) have become a significant public health concern requiring urgent action [42]. Saudi Arabia has the greatest RTA-associated mortality [43], contributing to 13% of the disability-adjusted life years (DALYs) in the Saudi population [44]. Saudi Arabia, the largest Arab state in Western Asia, is a high-income country with a population of over 27 million. Jizan has the highest population density, while Aljouf has the lowest [4]. Saudi Arabia’s transportation sector has significantly increased from 145,000 in 1970 to nearly 18 million, with approximately 800,000 vehicles imported annually [45]. Over the past two decades, KSA has recorded 86,000 deaths and 611,000 injuries in road traffic accidents, with 7% of injuries resulting in permanent disability [46].

Our study found a decrease in injuries in 2020 due to the Saudi government’s nationwide 11 h curfew in response to the COVID-19 pandemic. The curfew, which lasted from 7 p.m. to 6 a.m., restricted outdoor activities and travel between cities. The curfew was lifted in May 2020, coinciding with a campaign titled “We All Return Cautiously” [47]. A decrease in 2022 incidents was seen, possibly due to improved traffic law enforcement and increased adherence to safe driving practices [48]. The Saudi Arabian government is working towards Vision 2030 to improve road safety, with the Saudi Ministry of Interior aiming to reduce fatalities to six per 100,000 individuals [46]. Saudi Arabia’s multi-sectoral efforts have led to a 36% and 21% reduction in road traffic fatalities and injuries from 2017 to 2021 [49].

The study found that the mean age of patients with injury was 33–34 years, with variable disability prevalence, from low for ≥65 to high for 25–34, 35–44, and 18–24 years. Glèlè-Ahanhanzo et al. [50] reported that young, working-age people (average age: 30 years) are more likely to have disabilities in road traffic accidents than other age groups. Similarly, Abedzadeh-Kalahroudi et al. [51] showed that 56.5% of patients were under the age of 35 and were of working age. Disability caused by injuries is linked to financial and social problems resulting from challenges in finding a job. Prior studies conducted in Saudi Arabia yielded comparable findings. The average age recorded was 30.13 years, 30.7 years, and 32.57 years, according to Alslamah et al. [52], Algahtany [53], and Al Babtain et al. [54], respectively. AbdelRazik et al. [23] reported that the age of 38% of cases was 20 to 30 years, and 23.3% were 30 to 40 years. Shults et al. [32] study on disability prevalence of motor vehicle crashes in the USA found varying results across age groups, with a low of 375 per 100,000 for 18–24 years old and a high of 939 per 100,000 for 55–64 years old. The study found no significant link between patient age and high disability grade. However, patients aged 25–34 years were at higher risk. This was in disagreement with Abedzadeh-Kalahroudi et al. [51], who reported that older patients had higher disability scores while young patients had lower scores. A high percentage of injuries with a high grade of disability among the young age group in our study indicates the possible explanation for driving carelessly and with relatively little concern for road traffic instructions, as evidenced by reports [28]. 

The study found that the majority of patients were male, with a decline in percentage over time. However, there was a significant difference compared to females. It is important to note that only males were allowed to operate motor cars in Saudi Arabia for many years. Since June 2019, women have been granted the right to drive. Consequently, prior studies exhibited a greater proportion of male participants compared to female participants. Alslamah et al. [52] included 59.1% of the cases as men, whereas Algahtany [53], AbdelRazik et al. [23], Al Babtain et al. [54], Yagoub et al. [55], Gorge et al. [56], and Ahmed et al. [57] studies included 87.28%, 91%, 97.82%, 100%, 88%, and 74.02%, respectively. A previous study in the Aljouf region in 2019 [58] reported that road traffic accidents were the most common type of trauma, with males being more likely to have trauma due to their higher likelihood of engaging in violent activities and work stress. Similar results were reported in other countries: 69.8% in Kenya in 2013 [59], between 71.7% and 77% in Ethiopia in 2015 [60,61], 82% in Yemen in 2018 [62], and 79.36% in Benin in 2018 [50]. Peden [63] reported that the burden of road traffic injuries in terms of DALYs by gender, morbidity rates for males are considerably higher than those for females, which is probably related to both exposure and risk-taking behaviour.

Our research found that one injury was the most common type, and the occurrence of more than one injury declined over the years [50]. Alotaibi et al. [20] found a higher percentage of single fractures than multiple ones. However, Aloudah et al. [25] and Sonbol et al. [28] reported higher bilateral and multiple-site fractures. In our study, patients with three or more injuries were at risk of developing high grades of disability. Abedzadeh-Kalahroudi et al. [51] found that patients with multiple injuries had higher disability scores, attributed to a higher injury severity score (ISS). Most cases involved extremities, with right-side injuries being more common. The most common areas affected were the head and neck region, thorax, and lumbosacral spine. Hou et al. [17] and Adili et al. [64] found that road traffic accidents (RTAs) often result in fractures in the lower limbs, followed by upper limb and craniofacial region fractures. Chalya et al. [65] stated that road traffic accidents result in 60.5% of arm and leg injuries. In Saudi Arabia, the upper and lower extremities were the most frequently injured body parts (31–38%) in motor vehicle accidents [66,67,68]. Alotaibi et al. [20] revealed that the most frequent recorded injury was fractures of the lower limbs (49%), followed by fractures of the upper limbs (28%), pelvic fractures (11%), and spinal fractures (10%) among victims of road traffic accidents in Aseer region, Saudi Arabia. Injuries to the extremities from road crashes are typically not fatal, but they are a significant contributor to disability. Each year, 6–7% of those injured in road accidents in KSA are released with lasting disabilities [45]. 

Head and neck injury analysis showed that head trauma is the most common injury, affecting the skull base and parietal bone. The nasal bone is most affected in maxillofacial fractures, with subaxial cervical fractures more common. Similar studies in Saudi Arabia revealed that parietal and temporal bones were the most frequently seen craniofacial fractures [69,70]. Modi et al. [71] and Arora and Khajuria [72] found that parietal and temporal bones were the most commonly fractured skull bones in RTAs. The common causes of maxillofacial fractures are RTAs; when patients sustain moderate to severe maxillofacial injuries, they often develop disabilities and require long-term treatment [73]. Hitosugi et al. [73] reported that nasal fractures comprised 38.5%, maxillary fractures 25.6%, mandible fractures 17.9%, zygomatic fractures 10.3%, and orbital fractures 7.7%. Our study confirmed the prevalence of nasal fractures but found a slightly higher percentage of orbital fractures. The incidence of cervical spinal fractures varies in the literature. Gupta et al. [74] reported that the most frequent traumatic fracture occurred at C_2_. Their study indicated that car drivers were the most common victims. However, Wang et al. [75] reported that the most common fractures among motorcycle drivers were at C_3_–C_7_. Alkhathlan et al. [27] reported that the most common level of cervical spinal fractures was at C_6_. The studies differ due to factors like the type of vehicle in Wang et al. [75], the kind of trauma [76], the velocity of the vehicle during the collision, and preventive actions like using seat belts [27].

Middle ribs were most frequently fractured, followed by lower ribs. Multiple rib fractures were common, with right-sided fractures being the most common. The last thoracic vertebra is the most liable. The first and second lumbar vertebrae were the most affected. RTA is the primary cause of chest blunt trauma, leading to rib fractures, with the middle ribs being most commonly fractured (76.33%) [77]. The study found that the prevalence of upper rib fractures, particularly the first one, decreases due to its deep placement and protection. Indirect trauma, such as sudden forward head, neck, and trunk movement, often causes hyperextension of the neck and violent contraction of sternocleidomastoid and scalene muscles. The bending strain causes a fracture behind the attachment of the scalene medius muscle [77,78]. The lower region of the thoracic spine is the most susceptible to fractures, with 60% to 70% occurring between T11 and L2 being biomechanically vulnerable to stress [79]. Most vertebral fractures occur without spinal cord injury, with neurological injuries accounting for 14–38% of all fractures [80], explaining the low incidence of major musculoskeletal disability in our study as quadriplegia.

Analysis of individual upper limb injuries revealed that the right side is the most common side for upper limb injuries, with long bone fractures being the most common, especially the humerus. Dislocations were common for the shoulder, elbow, carpometacarpal, and interphalangeal. Severe injuries like distal forearm amputation and quadriplegia were the least common. Yang et al. [81] reported that the most common fracture sites were the humerus and radius, followed by the ulna. Similar to previous reports documented by Joeris et al. [82]. Haris et al. [83] found that radius and humerus fractures had the highest incidence rates of 10.5% and 10%, respectively. In comparison, phalanges–metacarpal bones had the lowest rate of 0.5%. Shults et al. [32] reported that limb paralysis constitutes 4.0% of all motor vehicle-related chronic health conditions. Alghnam et al. [3] reported that amputation accounts for 0.79% of patients in Saudi Arabia undergoing long-term rehabilitation after motor vehicle crashes. However, there is no definitive consensus on this issue due to the diverse nature of injuries, medical facility admission protocols, and demographics.

Regarding lower limb injuries, the right side was the most common, with long bone fractures being the most common, with the femur being the most prevalent, followed by the tibia and fibula. Pelvic bones like the acetabulum and hip bone fractures were also reported. Dislocation was less common in the pelvis and interphalangeal limbs than in the upper limbs. Metatarsal bones were more likely to be fractured. Severe injuries, such as amputation and quadriplegia, were the least common in association with the upper limb. The preponderance of lower extremity fractures in this study is similar to the findings reported by Chigblo et al. [84] and Omoke and Ekumankama [85]. As per Almansouf et al. [30], femoral fractures were the most common type of lower limb fractures after motor vehicle accidents (31.5%), primarily observed in pedestrians involved in road accidents, as they require significant energy to break. Middle-shaft femoral fractures are prevalent in young men, particularly on the right side, and are more common in the younger age group, the age group with the highest reckless driving rates [86]. Half of major joint dislocations involve shoulder dislocations, with anterior dislocations being the most common. The unstable shoulder joint is due to the shallow glenoid cavity [87,88].

The injury severity score (ISS) analysis showed that patients exhibiting moderate ISS were most prevalent, followed by mild ISS, severe ISS, and very severe ISS. Agudelo-Ledezma et al. [89] reported that patients exhibiting ISS < 25 were most common in traffic accidents. The most prevalent disability score was grade 1, followed by DS2, DS3, and DS4, with significant differences in disability grades each year. Abedzadeh-Kalahroudi et al. [51] demonstrated that 72.4% of participants experienced mild or moderate disability one month after trauma, with a link between high disability grades and trauma severity, with patients with severe, severe, and moderate disabilities at higher risk. Abedzadeh-Kalahroudi et al. [51] reported that patients with severe and very severe trauma had higher disability scores than those with ISS < 16. Bull [41] reported that all severe (DS 4) cases had an ISS of at least 25. Several studies have shown that functional outcome is related to ISS [90,91].

The findings of this study could help healthcare planning, resource allocation, and targeted preventive measures in the Aljouf region. It is appropriate to advocate for primary prevention of MVA dangers in order to modify young people’s driving behaviours, mainly via mass media campaigns. Health-related information may be shown in public areas such as schools and shopping malls. Appropriate countermeasures against drivers who engage in unsafe driving behaviours should be devised to reduce the magnitude of road traffic incidents. To minimise the frequency of traffic accidents, alcohol intake tests, drug use, seat belt use, driving speed, driver health, and vehicle maintenance conditions should also be periodically checked [46]

In conclusion, this is the first study highlighting musculoskeletal disabilities in the Aljouf region, Saudi Arabia, following MVAs. 

In our study, severe musculoskeletal disabilities were rare, but fractures were the most common following MVAs. The majority of cases involved extremities, with right-sided injuries being more common. The most common areas affected were the head and neck region, thorax, and lumbosacral spine. Long-term disability injuries require significant medical attention. It is important to conduct studies on patients who are treated in outpatient facilities to identify those at higher risk of becoming disabled following MVAs. By implementing more intensive medical treatment and rehabilitation, it may be possible to improve the long-term prognosis for patients with less severe acute injuries. The economic burden within the main workforce, i.e., the young population (20–39 years), is dire given the high number of injuries related to road accidents. As a result, this economically active age group is at risk of high morbidity and mortality due to RTAs unless several road regulations are implemented. Therefore, further investigation into minimising RTA from an economic standpoint gives merit to the country, and, more importantly, to the young cohort’s lives.

Further research should explore more variables and factors affecting the injury type, such as high-velocity impacts. Moreover, similar regional research is needed to compare and raise awareness. Additionally, these studies could provide insight into designing vehicles to lower the impact of a crash, lowering acute injuries that lead to long-term disability.

## Figures and Tables

**Table 1:** Demographic characteristics of the patients with musculoskeletal disabilities following motor vehicle accidents (*n* = 409).

Variables	The Year 2020		Year 2021		Year 2022		*p*-Value between Years
	N (%) Mean ± SD	*x*^2^ *(p*-Value)	N (%) Mean ± SD	*x*^2^ *(p*-Value)	N (%) Mean ± SD	*x*^2^ *(p*-Value)	
**Number of patients**	88		168		153		
**Age**	34.86 ± 10.32		34.27 ± 12.22		33.97 ± 12.19		0.84
**Age group (years)**		35.41 (<0.001 ^^^)		81.07 (<0.001 ^^^)		71.04 (<0.001 ^^^)	0.06
18–24	13 (14.8)		46 (27.4)		39 (25.5)		
25–34	36 (40.9)		40 (23.8)		53 (34.6)		
35–44	23 (26.1)		52 (31.0)		29 (19.0)		
45–54	13 (14.8)		23 (13.7)		22 (14.4)		
55–64	3 (3.4)		4 (2.4)		7 (4.6)		
≥65	0 (0.0)		3 (1.8)		3 (2)		
**Gender**		55.68 (<0.001 ^^^)		72.02 (<0.001 ^^^)		54.12 (<0.001 ^^^)	
Male	79 (89.8)		139 (82.7)		122 (79.7)		0.13
Female	9 (10.2)		29 (17.3)		31 (20.3)		
**Nationality**		20.05 (<0.001 ^^^)		54.86 (<0.001 ^^^)		49.47 (<0.001 ^^^)	0.65
Saudi	65 (73.90)		132 (78.6)		120 (78.4)		
Non-Saudi	23 (26.1)		36 (21.4)		33 (21.6)		

*x*^2^ = chi-square; ^^^ *p* value < 0.001 = statistically significant chi-square test.

**Table 2:** Injury characteristics of patients (*n* = 409).

Variables	Year 2020		Year 2021		Year 2022		*p*-Value between Years
	N (%) Mean (±SD)	*x*^2^ *(p*-Value)	N (%) Mean (±SD)	*x*^2^ *(p*-Value)	N (%) Mean (±SD)	*x*^2^ *(p*-Value)	
**Number of injuries**	88	86.27 (<0.001 ^^^)	168	151.00 (<0.001 ^^^)	153	184.75 (<0.001 ^^^)	0.52
One	70 (79.6)		130 (77.4)		130 (85.0)		
Two	14 (15.9)		30 (17.8)		17 (11.1)		
Three or more	4 (4.5)		8 (4.8)		6 (3.9)		
**Site of injury**		24.07 (<0.001 ^^^)		41.92 (<0.001 ^^^)		48.50 (<0.001 ^^^)	0.87
Head and neck (including cervical spine)	19 (21.6)		44 (26.2)		31 (20.3)		
Thorax (including thoracic spine)	18 (20.5)		41 (24.4)		26 (17)		
Spine (lumbosacral)	6 (6.8)		9 (5.4)		10 (6.5)		
Upper extremity (including shoulder girdle)	34 (38.6)		62 (36.9)		62 (40.5)		
Lower extremity (including pelvic girdle)	32 (36.4)		60 (35.7)		53 (34.6)		
**Trauma Severity (ISS)**	11.82 ± 7.71	32.52 (<0.001 ^^^)	11.23 ± 6.42	127.00 (<0.001 ^^^)	12.16 ± 7.28	35.89 (<0.001 ^^^)	0.04 ^
Mild, ISS < 9	17 (19.3)		24 (14.3)		32 (20.9)		
Moderate, ISS = 9–15	43 (48.9)		103 (61.3)		64 (41.8)		
Severe, ISS= 16–24	22 (25)		34 (20.2)		44 (28.8)		
Very severe, ISS ≥ 25	6 (6.8)		7 (4.2)		13 (8.5)		
**Disability score**		68.64 (<0.001 ^^^)		59.57 (<0.001 ^^^)		71.60 (<0.001 ^^^)	0.003 ^^
DS1	54 (61.4)		102 (60.7)		74 (48.4)		
DS2	11 (12.5)		42 (25.0)		45 (29.4)		
DS3	20 (22.7)		24 (14.3)		33 (21.6)		
DS4	3 (3.4)		0 (0.0)		1 (0.7)		
**Hospital admission**		0.41 (ns)		130.38 (<0.001 ^^^)		1.47 (ns)	<0.001 ^^^
Yes	47 (53.4)		158 (94)		84 (54.9)		
No	41 (46.6)		10 (6)		69 (45.1)		
**GCS**	14.44 ± 2.26		14.95 ± 0.56		14.53 ± 2.57		0.07

ISS = injury severity score, GCS = Glasgow coma scale, DS = disability Score, *x*^2^ = chi-square. ns = non-significant. ^ *p* value < 0.05, ^^ *p* value < 0.01, ^^^ *p* value < 0.001 = statistically significant chi-square test.

**Table 3:** Individual head and neck region injury.

Variables	Year 2020		Year 2021		Year 2022		*p*-Value between Years
	N (%)	*x*^2^ *(p*-Value)	N (%)	*x*^2^ *(p*-Value)	N (%)	*x*^2^ *(p*-Value)	
**Skull bones**	N = 2	0.00 (ns)	N = 25	29.56 (<0.001 ^^^)	N = 12	1.33 (ns)	0.10
The base of the skull with temporal bone	1 (50)		3 (12)		4 (33.3)		
Parietal bone	1 (0.0)		2 (8)		0 (0.0)		
Occipital bone	0 (0.0)		2 (8)		0 (0.0)		
Head trauma	1 (50.0)		18 (72)		8 (66.7)		
**Facial bones**	N = 11	9 (<0.05 ^)	N = 12	1.00 (ns)	N = 28	26.58 (<0.001 ^^^)	0.25
Orbital bone	1 (9.1)		2 (16.7)		5 (17.86)		
Nasal bone	7 (63.6)		3 (25.0)		14 (50.0)		
Zygomatic bone	0 (0.0)		2 (16.7)		1 (3.6)		
Maxillary bone	0 (0.0)		1 (8.2)		4 (14.3)		
Mandible	1 (9.1)		2 (16.7)		2 (7.2)		
Multiple facial bones	2 (18.2)		2 (16.7)		2 (7.2)		
**Cervical vertebra**	N = 5	1.80 (ns)	N = 7	1.29 (ns)	N = 11	2.27 (ns)	0.94
Upper cervical fracture (C1-C2)	1 (20.0)		2 (28.6)		3 (27.3)		
Subaxial cervical fracture (C3-C7)	4 (80.0)		5 (71.4)		8 (72.7)		

*x*^2^ = chi-square, ns = non-significant. ^ *p* value < 0.05, ^^^ *p* value < 0.001 = statistically significant chi-square test.

**Table 4:** Individual thoracic region injury.

Variables	Year 2020		Year 2021		Year 2022		*p*-Value between Years
	N (%)	*x*^2^ *(p*-Value)	N (%)	*x*^2^ *(p*-Value)	N (%)	*x*^2^ *(p*-Value)	
**Location of fractured ribs**	N = 10	1.6 (ns)	N = 28	19.14 (<0.001 ^^^)	N = 28	22.36 (<0.001 ^^^)	0.16
Upper (1st–3rd ribs)	0 (0.0)		6 (21.4)		2 (7.1)		
Middle (4th–9th ribs)	7 (70.0)		20 (71.4)		21 (75.0)		
Lower (10th–12th ribs)	3 (30.0)		2 (7.1)		5 (17.9)		
**Number of fractured ribs**							
1–2	2 (20.00)	3.6 (ns)	9 (36.0)	8.72 (<0.05 ^)	7 (26.9)	22.36 (<0.01 ^^)	0.04 ^
3–5	8 (80.0)		14 (56.0)		17 (65.4)		
≥6	0 (0.0)		2 (8.0)		2 (7.7)		
**Side of fractured ribs**		5.33 (0.02)		13.37 (<0.001 ^^^)		15.13 (<0.001 ^^^)	0.99
Right	10 (83.3)		23 (85.2)		27 (84.4)		
Left	2 (16.7)		4 (14.8)		5 (15.6)		
**Thoracic vertebra**	N = 7	3.86 (ns)	N = 22	4.91 (ns)	N = 11	1.00 (ns)	0.51
1st thoracic vertebra	0 (0.0)		0 (0.0)		2 (18.2)		
4th thoracic vertebra	0 (0.0)		3 (13.6)		0 (0.0)		
5th thoracic vertebra	1 (14.3)		4 (18.2)		2 (18.2)		
6th thoracic vertebra	0 (0.0)		3 (13.6)		0 (0.0)		
7th thoracic vertebra	1 (14.3)		3 (13.6)		0 (0.0)		
8th thoracic vertebra	0 (0.0)		2 (9.1)		0 (0.0)		
9th thoracic vertebra	0 (0.0)		1 (4.5)		0 (0.0)		
10th thoracic vertebra	0 (0.0)		1 (4.5)		0 (0.0)		
11th thoracic vertebra	1 (14.3)		0 (0.0)		3 (27.3)		
12th thoracic vertebra	4 (57.1)		5 (22.7)		4 (36.4)		

*x*^2^ = chi-square, ns = non-significant. ^ *p* value < 0.05, ^^ *p* value < 0.01, ^^^ *p* value < 0.001 = statistically significant chi-square test.

**Table 5:** Lumbosacral spine.

Variables	Year 2020		Year 2021		Year 2022		*p*-Value between Years
	N (%)	*x*^2^ *(p*-Value)	N (%)	*x*^2^ *(p*-Value)	N (%)	*x*^2^ *(p*-Value)	
**Lumbar vertebra**	N = 7	0.14 (ns)	N = 9	1.22 (ns)	N = 6	2.67 (ns)	0.35
1st lumbar vertebra	4 (57.1)		3 (33.3)		5 (83.3)		
2nd lumbar vertebra	3 (42.9)		3 (33.3)		1 (16.7)		
3rd lumbar vertebra	0 (0.0)		2 (22.2)		0 (0.0)		
5th lumbar vertebra	0 (0.0)		1 (11.1)		0 (0.0)		

*x*^2^ = chi-square, ns = non-significant.

**Table 6:** Individual upper limb injury.

Variables	Year 2020		Year 2021		Year 2022		*p*-Value between Years
	N (%)	*x*^2^ *(p*-Value)	N (%)	*x*^2^ *(p*-Value)	N (%)	*x*^2^ *(p*-Value)	
**Side of the fractured limb**	N = 42	4.67 (0.03)	N = 68	28.47 (<0.001 ^^^)	N = 69	8.23 (<0.01 ^^)	0.07
Right	28 (66.7)		56 (82.4)		46 (66.7)		
Left	14 (33.3)		12 (17.6)		23 (33.3)		
**Upper limb region**	N = 43	60.09 (<0.001 ^^^)	N = 68	29.35 (<0.01 ^^)	N = 70	66.80 (<0.001 ^^^)	0.25
Clavicle	5 (11.6)		8 (11.8)		12 (17.1)		
Scapula	3 (7.0)		0 (0.0)		4 (5.7)		
Humerus	15 (34.9)		17 (25.0)		19 (27.1)		
Radius	7 (16.3)		10 (14.7)		14 (20.0)		
Ulna	5 (11.6)		7 (10.3)		3 (4.3)		
Carpal bones	1 (2.3)		1 (1.5)		2 (2.9)		
Metacarpal bones	1 (2.3)		7 (10.3)		6 (8.6)		
Phalanges	1 (2.3)		4 (5.9)		1 (1.4)		
Shoulder dislocation	1 (2.3)		9 (13.2)		5 (7.1)		
Elbow dislocation	1 (2.3)		3 (4.4)		2 (2.9)		
Carpometacarpal dislocation	1 (2.3)		0 (0.0)		0 (0.0)		
Interphalangeal dislocation	1 (2.3)		0 (0.0)		1 (1.4)		
**Amputation distal forearm**	0 (0.0)		2 (2.9)		0 (0.0)		
**Quadriplegia**	1 (2.3)		0 (0.0)		1 (1.4)		

*x*^2^ = chi-square. ^^ *p* value < 0.01, ^^^ *p* value < 0.001 = statistically significant chi-square test.

**Table 7:** Individual lower limb injury.

Variables	Year 2020		Year 2021		Year 2022		*p*-Value between Years
	N (%)	*x*^2^ *(p*-Value)	N (%)	*x*^2^ *(p*-Value)	N (%)	*x*^2^ *(p*-Value)	
**Side of fractured limb**	N = 38	0.95 (ns)	N = 84	58.33 (<0.001 ^^^)	N = 75	4.81 (<0.05 ^)	<0.001 ^^^
Right	22 (57.9)		77 (91.7)		47 (62.7)		
Left	16 (42.1)		7 (8.3)		28 (37.3)		
**Lower limb region**	N = 41	17.02 (ns)	N = 86	29.39 (<0.001 ^^^)	N = 76	73.58 (<0.001 ^^^)	<0.01 ^^
Acetabulum	4 (9.8)		11 (12.8)		7 (9.2)		
Hip bone	2 (4.9)		5 (5.8)		6 (7.9)		
Femur	7 (17.1)		20 (23.3)		21 (27.6)		
Patella	4 (9.8)		0 (0.0)		1 (1.3)		
Tibia	6 (14.6)		14 (16.3)		12 (15.8)		
Fibula	6 (14.6)		13 (15.1)		9 (11.8)		
Tarsal bones	1 (2.4)		2 (2.3)		3 (3.9)		
Metatarsal bones	3 (7.3)		8 (9.3)		6 (8.6)		
Phalanges	0 (0.0)		0 (0.0)		2 (2.6)		
Pelvic fracture	3 (7.3)		7 (8.1)		4 (5.3)		
Pelvic dislocation	1 (2.4)		0 (0.0)		3 (3.9)		
Interphalangeal dislocation	1 (2.4)		0 (0.0)		1 (1.3)		
Ankle trauma	0 (0.0)		3 (3.5)		4 (5.3)		
**Amputation lower limb**	1 (2.4)		0 (0.0)		0 (0.0)		
**Quadriplegia**	2 (4.9)		0 (0.0)		1 (1.3)		

*x*^2^ = chi-square, ns = non-significant. ^ *p* value < 0.05, ^^ *p* value < 0.01, ^^^ *p* value < 0.001 = statistically significant chi-square test.

**Table 8:** Correlation between age group, gender, and injury site, and disability index.

Variables	DS1 (N = 230)	DS2 (N = 98)	DS3 (N = 77)	DS4 (N = 4)	*x*^2^ *(p*-Value) Cramer’s V
	N (%)	N (%)	N (%)	N (%)	
**Age group (years)**					19.507 (ns)
18–24	59 (60.2)	26 (26.5)	13 (13.3)	0 (0.0)	
25–34	63 (48.8)	34 (26.4)	29 (22.5)	3 (2.3)	
35–44	63 (60.6)	16 (15.4)	24 (23.1)	1 (1.0)	
45–54	36 (62.1)	13 (22.4)	9 (15.5)	0 (0.0)	
55–64	7 (50.0)	6 (42.9)	1 (7.1)	0 (0.0)	
≥65	2 (33.3)	3 (50.0)	1 (16.7)	0 (0.0)	
**Gender**					5.736 (ns)
Male	189 (55.6)	77 (22.6)	70 (20.6)	4 (1.2)	
Female	41 (59.4)	21 (30.4)	7 (10.1)	0 (0.0)	
**Site of injury**					9.191 (ns)
Head and neck (including cervical spines)	44 (46.7)	34 (36.2)	15 (16.0)	1 (1.1)	
Thorax (including thoracic spines)	60 (70.6)	17 (20.0)	8 (9.4)	0 (0.0)	
Spine (lumbosacral)	13 (52.0)	7 (28.0)	5 (20.0)	0 (0.0)	
Upper extremity (including shoulder girdle)	91 (49.2)	34 (18.4)	32 (17.3)	1 (0.5)	
Lower extremity (including pelvic girdle)	57 (39.3)	35 (24.1)	51 (35.2)	2 (1.4)	

DS = disability score, *x*^2^ = chi-square, ns = non-significant.

**Table 9:** Relative risk, odd ratio at 95% interval for disability DS3 and DS4 risk factors.

Variables	Odds Ratio (95% CI)	Relative Risk (95% CI)	*p*-Value Fisher’s Exact Test
**Sex**			
Female	Ref	Ref	Ref
Male	2.46 (1.09–5.79)	2.15 (1.08–4.46)	0.03 ^
**Age (years)**			
≥65	Ref	Ref	Ref
18–24	0.76 (0.10–9.64)	0.80 (0.20–4.62)	0.59 (ns)
25–34	1.65 (0.21–19.99)	1.49 (0.41–8.40)	0.99 (ns)
35–44	1.58 (0.20–19.32)	1.44 (0.39–8.17)	0.99 (ns)
45–54	0.92 (0.13–11.91)	0.93 (0.22–5.50)	0.99 (ns)
55–64	0.38 (0.02–8.67)	0.43 (0.05–3.85)	0.52 (ns)
**Trauma Severity(ISS)**			
Mild, ISS < 9	Ref	Ref	Ref
Moderate, ISS = 9–15	∞ (1.22–∞)	∞ (1.22–∞)	0.02 ^
Severe, ISS = 16–24	∞ (20.90–∞)	∞ (2.00–∞)	<0.0001 ^^^
Very Severe, ISS ≥ 25	∞ (39.81–∞)	∞ (2.00–∞)	<0.0001 ^^^
**Number of injuries**			
One	Ref	Ref	Ref
Two	2.26 (1.23–4.15)	1.87 (1.18–2.85)	0.01 ^
Three or more	3.18 (1.23–8.59)	2.33 (1.17–3.98)	0.02 ^
**Site of injury**			
Spine (lumbosacral)	Ref	Ref	Ref
Thorax (including thoracic spines)	0.41 (0.13–1.27)	0.47 (0.18–1.28)	0.16 (ns)
Head and neck (including cervical spines)	0.83 (0.27–2.27)	0.86 (0.38–2.14)	0.77 (ns)
Upper extremity (including shoulder girdle)	1.07 (0.37–2.78)	1.06 (0.50–2.50)	0.99 (ns)
Lower extremity (including pelvic girdle)	2.26 (0.81–5.74)	1.80 (0.88–4.17)	0.16 (ns)

ISS = injury severity score, ns = non-significant, ^ *p* value < 0.05, ^^^ *p* value < 0.001 = statistically significant chi-square test.

## Data Availability

Available upon reasonable request.
